# Robust transcriptomic signatures of Alzheimer’s disease progression: validated explainable AI approach

**DOI:** 10.1038/s41598-026-47879-8

**Published:** 2026-05-19

**Authors:** Reham A. Shafik, Yasmine M. Afify, Nagwa Badr, Mahmoud Mounir

**Affiliations:** Information Systems, Faculty of Computer and Information Sciences, Ain Shams University, Cairo, Egypt

**Keywords:** Braak-staging, Gene expression, Transcriptomics, Brain regions, XGBoost, SHAP, SMOTE, Explainable AI, Biomarkers, Biomarkers, Computational biology and bioinformatics, Neuroscience

## Abstract

The lack of validated stage-specific biomarkers hampers the understanding of Alzheimer’s disease (AD) progression and clinical translation. Current transcriptomic methods often produce unstable results with limited stage discrimination. We aimed to develop an explainable machine learning pipeline to identify robust, interpretable gene signatures linked to distinct AD neuropathological stages. We analyzed multi-region transcriptomic data from the MSBB cohort using a multi-class XGBoost-SHAP-driven framework. Class imbalance was addressed using SMOTE, and model robustness was assessed through cross-validation and permutation-based validation. Our pipeline accurately classified Early, Mid, and Late Braak stages and achieved accurate Braak stage classification (regional ROC AUCs up to 0.76). The method identified a concise set of high-confidence, stage-specific genes, showing minimal signature overlap (~ 1.7%). Key validated novel candidate biomarkers included *ARX* (Early), *MKNK2* (Mid), and *SLC25A16/NEURL1B* (Late), linked to GABAergic, inflammatory, mitochondrial, and synaptic pathways. This explainable framework overcomes key limitations of conventional analyses, providing a stable, interpretable gene signature for AD staging. It establishes a robust method for transcriptional biomarker discovery and offers new biological insight into AD progression, highlighting potential stage-specific therapeutic targets.

**Supplementary Information:**

The online version contains supplementary material available at 10.1038/s41598-026-47879-8.

## Background

Alzheimer’s disease (AD) represents one of the most significant challenges in modern healthcare, with its prevalence expected to triple by 2050 as global populations age^1^. As a progressive neurodegenerative disorder, AD follows a characteristic neuropathological trajectory described by Braak staging, where neurofibrillary tangles progressively spread through specific brain regions from transentorhinal regions (Braak I–II) to limbic areas (Braak III–IV) and ultimately throughout neocortical regions (Braak V–VI)^2^. Understanding the molecular drivers of this progression is crucial for developing effective therapeutic interventions and reliable biomarkers.

Alzheimer’s disease exhibits both temporal and spatial heterogeneity. Stage-specific biomarkers capture the temporal dynamics of disease progression, identifying molecular processes active at Early, Mid, or Late Braak stages. Region-resolved biomarkers reveal spatial differences in vulnerability across brain regions, highlighting why some areas are more susceptible than others. Both approaches are complementary: stage-specific biomarkers inform when pathological processes occur, while region-specific biomarkers indicate where they manifest. Combining temporal and spatial perspectives provides a comprehensive understanding of AD progression, enabling high-resolution mapping of transcriptional changes and biologically interpretable predictive models.

While peripheral biomarkers from blood and cerebrospinal fluid have shown promise for AD diagnosis^3^, they provide limited insights into the region-specific molecular mechanisms driving disease progression within the brain itself. AD neuropathology exhibits marked regional specificity, with vulnerable areas such as the entorhinal cortex and hippocampus affected early, while primary sensory and motor regions are relatively spared until late stages^4^. This spatial pattern suggests that local transcriptional programs may determine vulnerability or resilience. Moreover, blood-brain barrier permeability and differential gene expression between peripheral tissues and the central nervous system mean that blood-based signatures often fail to capture the brain’s complex regulatory networks^5^. Brain-region specific analysis therefore enables the identification of molecular cascades reflecting cell-autonomous vulnerabilities or cell-type specific responses to pathology.

The emergence of explainable AI (XAI) methods, particularly SHAP (SHapley Additive exPlanations), now enables researchers to move beyond “black box” predictions to understand which features drive model decisions^6^. This is particularly valuable in biomedical contexts where biological interpretability is as important as predictive accuracy.

In this study, we present a comprehensive machine learning framework that addresses these limitations through several key innovations: *Brain-Region Specific Focus*: We analyze gene expression data across multiple brain regions to identify region-specific transcriptional signatures of AD progression. *Stage-Wise Classification*: We group Braak stages into clinically meaningful categories (Early, Mid, Late) to identify genes specifically important at different disease phases. *Rigorous Validation Protocol*: We implement a multi-level validation strategy including cross-validation stability analysis, permutation-based significance testing and test set importance confirmation to ensure the reliability of identified biomarkers. *Comprehensive Visualization*: We employ heatmaps to visualize stage-specific important genes across brain regions and regional effectiveness charts to identify brain regions that are most informative for classifying each disease stage.

Our approach leverages XGBoost with SHAP explainability and addresses class imbalance through SMOTE oversampling. We validate the model performance using AUC and F1 scores for each Braak stage group and provide robust, interpretable gene importance rankings that have been rigorously validated across multiple testing paradigms.

This study aims to provide not just a predictive model, but a biologically interpretable framework for understanding the transcriptional dynamics of AD progression across different brain regions, contributing to the identification of novel therapeutic targets and stage-specific biomarkers.

The rest of the paper is structured as follows: “Related work” supports the background with the reviews of the existing literature on transcriptomic studies of AD, machine learning applications in biomarker discovery, and molecular correlates of Braak staging. “Methods and materials” details the used MSBB dataset, our preprocessing steps, the formulation of Braak stage groups, and our machine learning pipeline, including the XGBoost model, SMOTE balancing, and our rigorous gene validation protocol. “Results” presents the model’s performance in classifying Braak stages, identifies the high-confidence genes validated through our approach, and highlights the most informative brain regions for each stage through comprehensive visualizations. “Discussion” interprets the biological significance of our findings, places them in the context of existing literature, acknowledges the limitations of our study, and suggests directions for future research. Finally, “Conclusion” synthesizes the main findings of the study, considerations and suggested future works.

### Related work

The application of machine learning (ML) to transcriptomic data has significantly advanced our understanding of complex neurodegenerative diseases like Alzheimer’s disease (AD). Traditional transcriptomic studies have primarily relied on differential expression analysis to identify genes associated with disease states. For example, multiscale network analysis of oligodendrocytes in the ROSMAP dataset identified dysregulated genes involved in myelination, synaptic function, and immune response^7^, while single-nucleus RNA sequencing revealed cell-type-specific transcriptional changes in microglia and neurons across disease progression^8^. These approaches successfully highlighted candidate genes but often treated AD as a binary condition, overlooked stage-specific progression, and focused on associations rather than predictive relationships.

Machine learning methods have been applied to overcome these limitations by modeling complex, non-linear interactions between genes and disease phenotypes. Using RNA-seq data from the Mount Sinai Brain Bank (MSBB), prior studies identified molecular subtypes and subtype-specific biomarkers across brain regions, linking transcriptional heterogeneity to pathology^9^. Other approaches, such as AITeQ, demonstrated that compact, interpretable gene signatures can distinguish AD from controls using ensemble ML models^10^. However, these studies generally focus on molecular heterogeneity or binary classification and often lack rigorous validation for stage-specific biomarker stability.

Several studies have attempted to link gene expression to Braak staging, highlighting transcriptional changes across disease progression. Analyses made of the temporal cortex in the Mount Sinai cohort showed progressive dysregulation of synaptic and immune genes across Braak stages^11^, and deep learning-based severity indices from bulk RNA-seq captured non-linear transcriptional dynamics correlated with Braak stage and tau pathology^12^. While these approaches provide insights into molecular correlates of AD progression, they often rely on bulk tissue, linear correlations, or lack direct prediction of individual Braak stages, limiting reproducibility of stage-specific biomarkers.

Region-resolved biomarker discovery has also been explored using imaging-based ML models, such as SVR and ANN on amyloid-β PET data, demonstrating that regional pathology can inform cognitive decline^13^. Yet, these studies are often constrained by small sample sizes, lack external validation, and do not fully account for class imbalance or robust validation of explanatory biomarkers. Similarly, comparative studies highlight that peripheral blood biomarkers may track general neurodegeneration but fail to capture brain-specific transcriptional changes, underscoring the need to study brain tissue directly for reliable biomarker discovery^14,15^.

Finally, explainable AI (XAI) methods have enabled interpretable ML in neuroscience, allowing identification of features driving model decisions. Techniques like SHAP and Integrated Gradients have been used to localize biologically plausible regions influencing predictions and enhance interpretability of models^16^. However, most XAI applications in AD focus on neuroimaging rather than transcriptomics, leaving a gap for interpretable, brain-region- and stage-specific gene biomarker discovery—an objective addressed by our study.

#### Knowledge gaps and our contribution

The current literature reveals several significant knowledge gaps that our study addresses. First, while multiple studies have applied ML to AD transcriptomics, few have focused specifically on predicting Braak stage progression with appropriate handling of class imbalance. Second, existing explainable AI applications in AD have predominantly focused on neuroimaging data, with limited application to transcriptomics. Third, current biomarker validation practices often lack the multi-level approach necessary to ensure biological reliability and clinical relevance.

Our work makes four key contributions to address these gaps:

1. **Progression-aware modeling:**

We implement a stage-wise classification approach that models AD as a progressive continuum rather than a binary state.

2. **Comprehensive validation strategy:**

We introduce a comprehensive validation framework combining cross-validation stability analysis, permutation-based significance testing with test set importance confirmation.

3. **Robust handling of class imbalance:**

We address class imbalance through SMOTE oversampling, ensuring balanced performance across all disease stages.

4. **Region-resolved biomarker discovery:**

We provide region-specific biomarker identification through brain-region specific analysis and visualization.

By combining rigorous machine learning methodology with biological interpretability and multi-layered validation, our approach advances the field beyond simple classification toward clinically meaningful, stage-specific biomarker discovery.

## Materials and methods

In this study, we conducted a comprehensive transcriptomic analysis to investigate stage-specific molecular changes in Alzheimer’s disease. Our methodology integrates large-scale RNA-seq data from multiple brain regions with detailed clinical and neuropathological annotations, focusing on Braak staging as the primary outcome measure. Below, we outline the acquisition and preprocessing of gene expression data, integration of clinical metadata, feature engineering, predictive modeling framework, and multi-level validation strategies. This workflow enables robust identification and interpretation of stage-specific biomarkers while ensuring reproducibility and biological relevance across brain regions and disease progression stages.

### Data acquisition and preprocessing

To explore transcriptional changes across Alzheimer’s disease progression, we acquired large-scale RNA-seq data alongside detailed clinical and neuropathological metadata. Our preprocessing pipeline ensured consistent formatting, quality control, and normalization across multiple brain regions. This step also included mapping gene identifiers, aligning expression data with clinical annotations, and preparing the datasets for downstream predictive modeling. Careful attention to data integrity at this stage is critical for accurate modeling of Braak stage–specific molecular signatures.

#### MSBB dataset

Gene expression data (RNA-seq) and corresponding clinical metadata were obtained from the Mount Sinai Brain Bank (MSBB) study^17^. This post-mortem cohort is particularly valuable for studying AD progression as it includes individuals spanning the full spectrum of cognitive and neuropathological severity.

A total of 125 subjects were included in this study. Samples were categorized into Early (Braak 0–2, *n* = 33), Mid (Braak 3–4, *n* = 40), and Late (Braak 5–6, *n* = 51) stages. The cohort had a mean age of 84.13 ± 7.23 years, with a female predominance (72%). Detailed demographic and clinical characteristics of the cohort are summarized in (Table 1). The number of individuals per brain region, including overlapping subjects across regions, is summarized in (Table 2). The relatively balanced distribution across Braak stages supports the use of stratified cross-validation to preserve class proportions during model training.

**Table 1:** Cohort characteristics (Mount Sinai Brain Bank – MSBB).

Variable	Value
Total samples	125
Early stage (Braak 0–2)	33
Mid stage (Braak 3–4)	40
Late stage (Braak 5–6)	51
Female (%)	72.0%
Male (%)	28.0%
Mean age (± SD)	84.13 ± 7.23
Mean PMI (± SD)	361.02 ± 318.73
Mean brain pH (± SD)	6.37 ± 0.28

**Table 2:** Number of individuals per brain region in the study cohort.

Brain region	Number of individuals
Hipp	55
Cauda	52
Dors	57
IFG	53
ITG	58
MTG	58
PCC	58
PC	56
Puta	52
TP	58
Amyg	51
Total individuals	125

Our comprehensive analysis included eleven distinct brain regions that represent major anatomical structures implicated in Alzheimer’s disease progression: Hippocampus (Hipp), a core region for memory formation and one of the earliest affected sites; Caudate Nucleus (Caud) and Putamen (Puta), subcortical structures involved in cognitive and motor regulation; Amygdala (Amyg), critical for emotional processing and early pathological vulnerability; Inferior Temporal Gyrus (ITG) and Middle Temporal Gyrus (MTG), associated with semantic memory and language-related processing; Temporal Pole (TP), implicated in higher-order semantic and emotional integration; Posterior Cingulate Cortex (PCC), a central hub of the default mode network; Prefrontal Cortex (PC) and Dorsolateral Prefrontal Cortex (Dors), essential for executive and working memory functions; and Inferior Frontal Gyrus (IFG), involved in language production and social cognition. This extensive regional coverage enables a comprehensive spatial-temporal analysis of transcriptional changes across the entire Braak staging spectrum.

#### Clinical data integration and braak staging

The primary neuropathological outcome was the Braak score, a standardized staging system (ranging from zero to VI) for the progression of neurofibrillary tangles in Alzheimer’s disease. For each sample, the Braak score was extracted from the clinical metadata and used as the label for our predictive model.

Critical to our analysis was the precise integration of clinical neuropathological data with gene expression profiles. This was achieved by mapping samples from each brain region using ENTREZ GENE IDs as the universal identifier. The mapping process ensured that: each gene expression profile was accurately linked to its corresponding Braak stage, the regional analyses-maintained sample-specific clinical annotations and cross-region comparisons were biologically meaningful and consistent. This rigorous mapping protocol guaranteed that the transcriptional patterns learned by our model were directly associated with the appropriate stage of neurofibrillary tangle pathology for each specific brain region sample.

#### Data preprocessing

The analysis of high-dimensional gene expression data presents unique challenges, including technical noise, batch effects, and data distributions that can confound machine learning models. Effective preprocessing is therefore not merely a preliminary step but a critical component for ensuring biological signals is distinguished from artifacts, thereby enhancing the validity, reliability, and interpretability of the results.

A comprehensive preprocessing pipeline was implemented to ensure data quality and enhance downstream machine learning performance. The gene expression count matrix was first transposed to structure it as samples × genes. Low-variance genes (variance < 0.1) were filtered out to remove uninformative features, reducing dimensionality while preserving biological signals. To mitigate the influence of outliers, robust scaling was applied per gene using the interquartile range, followed by Z-score standardization to normalize each gene to zero mean and unit variance. Quality control metrics confirm successful normalization, with post-processing means approaching zero (mean of means ≈ 0) and variances approaching unity (mean of standard deviations ≈ 1). Finally, expression values for genes with duplicate ENTREZ identifiers were aggregated by mean calculation, ensuring unique gene representation in the final feature matrix. This rigorous preprocessing protocol produced optimized, region-specific datasets. The number of genes retained after preprocessing for each brain region is summarized in (Table 3).

**Table 3:** Gene counts after preprocessing by brain region.

Brain region	Initial genes	Genes after	Genes removed
Hipp	36,179	12,231	23,948
Caud	36,179	11,063	25,116
Dors	36,179	11,384	24,795
IFG	36,179	10,725	25,454
ITG	36,179	12,039	24,140
MTG	36,179	12,192	23,987
PCC	36,179	11,495	24,684
PC	36,179	10,824	25,355
Puta	36,179	11,881	24,298
TP	36,179	9346	26,833
Amyg	42,438	15,900	26,538

Number of genes measured per brain region before and after preprocessing, showing genes retained and removed after filtering and quality control.

### Problem formulation and label engineering

To model the progression of Alzheimer’s pathology, we framed the task as a supervised multi-class classification problem in which the objective is to predict the neuropathological stage of a sample based on its transcriptomic profile. Braak scores (0–VI), which quantify neurofibrillary tangle burden, were transformed into three clinically meaningful categories to reduce sparsity and improve class balance:

Class 0 (Early-stage): Braak 0–II.

Class 1 (Mid-stage): Braak III–IV.

Class 2 (Late-stage): Braak V–VI.

This grouping reflects well-established neuropathological transitions from initial entorhinal involvement to limbic spread and finally neocortical degeneration. Grouping also mitigates the severe class imbalance present in raw Braak stages while preserving clinically relevant boundaries.

Label engineering included aligning each gene expression sample with its corresponding Braak category, filtering out samples with missing or ambiguous staging information, and ensuring that labels were assigned independently for each brain region to maintain anatomical specificity. This formulation allows the model to learn transcriptional signatures characteristic of early, intermediate, and advanced stages of AD pathology.

### Machine learning pipeline

**Fig. 1:**
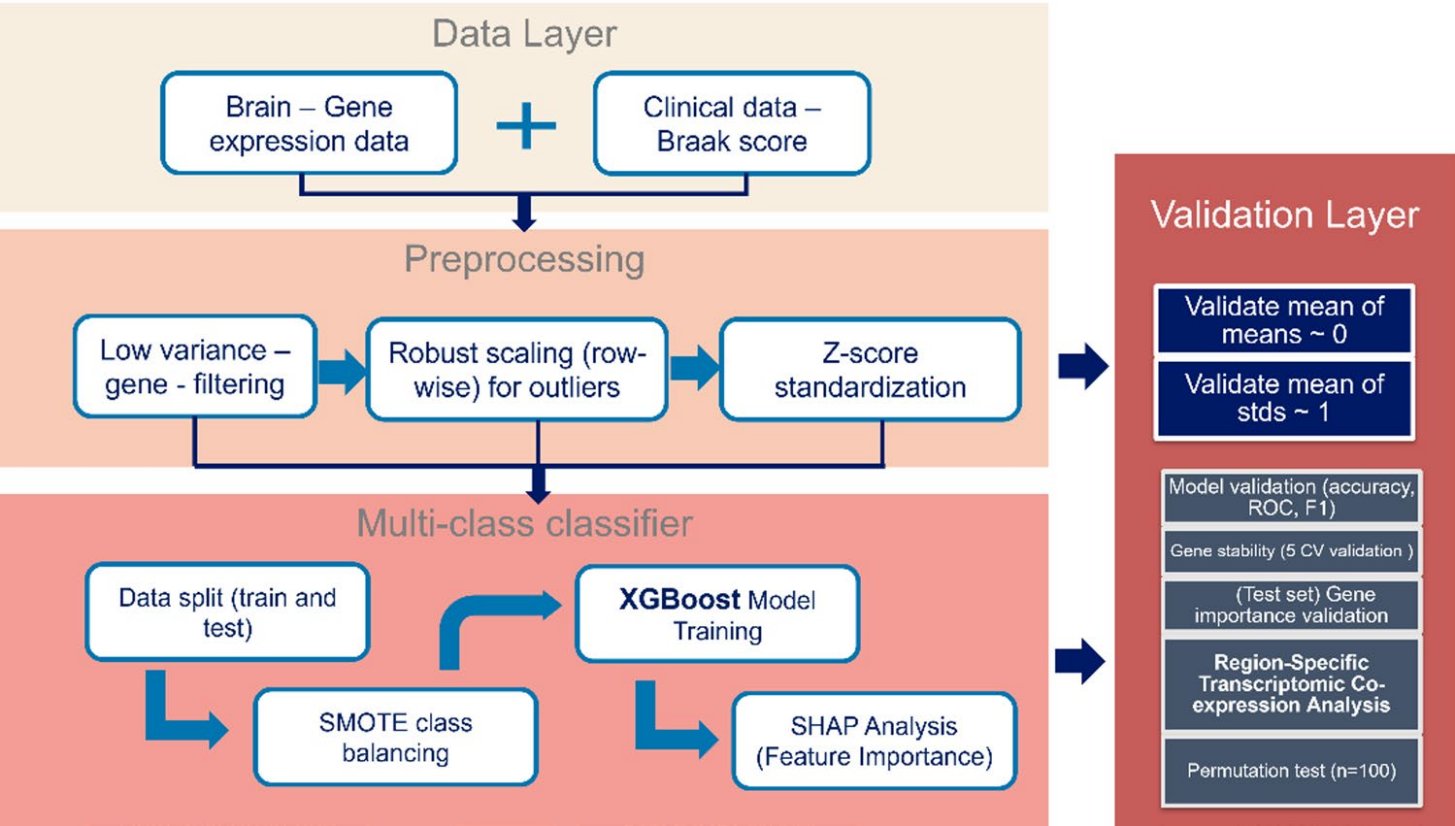
Architecture of the explainable ml pipeline for braak stage classification.

The overall architecture of our analytical pipeline, encompassing data preprocessing, model training, explainability, and validation, is illustrated in Fig. 1. This comprehensive framework was applied separately to each of the eleven brain regions to identify stage-specific transcriptional biomarkers.

We employed XGBoost (eXtreme Gradient Boosting), an ensemble tree-based algorithm known for its high performance and handling of complex non-linear relationships. The model was configured as a multi-class classifier (objective="multi: softprob”) with the following key hyperparameters to prevent overfitting and enhance generalization: n_estimators = 100, max_depth = 10, learning_rate = 0.005, subsample = 0.5, colsample_bytree = 0.5, and L1/L2 regularization (reg_alpha = 0.1, reg_lambda = 0.5).

XGBoost was used to train predictive models for Braak stage classification. After model evaluation on held-out test data, SHAP (SHapley Additive exPlanations) values were computed to quantify the contribution of each gene to model predictions, enabling biologically interpretable identification of stage-specific transcriptomic biomarkers.

While XGBoost provides native feature importance metrics based on split statistics such as information gain or gain frequency, these measures primarily provide a global ranking of features and may be biased toward variables frequently used in tree splits. In contrast, SHAP is grounded in cooperative game theory and calculates Shapley values that consistently attribute each feature’s contribution to individual predictions. This allows both global interpretation across the dataset and local interpretation at the sample level. In our framework, SHAP was therefore applied as a post-hoc explainability method on the trained XGBoost model to obtain more reliable and biologically interpretable estimates of gene importance across Braak stages and brain regions.

#### Data splitting and class imbalance handling

To ensure robust evaluation, the dataset was partitioned into a training set (70%) and a held-out test set (30%) using stratified sampling by Braak stage to preserve class distribution. The test set remained fully isolated during model development to provide an unbiased estimate of generalizability. To address class imbalance across Braak stages, SMOTE was applied only to the training data, generating synthetic samples for minority classes by interpolating existing instances. This balancing strategy prevents bias toward the majority class, ensuring strong F1 scores and consistent performance across Early, Mid, and Late stages. By exposing the model to sufficient examples of all stages, SMOTE supports reliable per-class and macro F1 scores measured on the untouched test set.

#### Model validation and performance metrics

Model performance was evaluated on the untouched test set. (results in Table 4) We reported multi-class metrics including overall accuracy, F1-score (macro-averaged and per-class), and the Area Under the Receiver Operating Characteristic Curve (AUC-ROC) for each Braak stage group.

**Table 4:** Model performance metrics across brain regions.

Brain region	Accuracy	ROC AUC (Macro)	F1-Score (Class 0)	F1-Score (Class 1)	F1-Score (Class 2)
PCC	0.5556	0.7596	0.6154	0.5333	0.5000
ITG	0.5000	0.7170	0.6667	0.4615	0.3636
IFG	0.3125	0.6528	0.4286	0.4000	0.000
Caud	0.3750	0.7014	0.4615	0.4000	0.2222
Hipp	0.4118	0.6730	0.5455	0.3077	0.4000
TP	0.5000	0.6898	0.5000	0.7143	0.2000
MTG	0.3889	0.6601	0.3333	0.4706	0.2857
Pref	0.3529	0.6151	0.3077	0.4615	0.2500
Puta	0.3750	0.5338	0.4000	0.4706	0.2000
Dors	0.3333	0.5295	0.3636	0.4286	0.1818
Amyg	0.3125	0.4939	0.3077	0.2000	0.4444

Performance metrics of XGBoost-SHAP models using transcriptomic data from each brain region. Brain Region indicates the anatomical region analyzed (e.g., PCC: Posterior Cingulate Cortex; ITG: Inferior Temporal Gyrus; IFG: Inferior Frontal Gyrus; Caud: Caudate; Hipp: Hippocampus; TP: Temporal Pole; MTG: Middle Temporal Gyrus; Pref: Prefrontal Cortex; Puta: Putamen; Dors: Dorsolateral Prefrontal Cortex; Amyg: Amygdala). Accuracy is the overall fraction of correctly classified samples across all stages. ROC AUC (Macro) is the area under the receiver operating characteristic curve, averaged across classes, indicating discriminative ability. F1-Score (Class 0/1/2) is the harmonic mean of precision and recall for each stage (0: Early, 1: Mid, 2: Late). Regions with high SHAP-based importance contribute strongly to stage predictions, with positive values pushing predictions toward a stage and negative values pushing away; low importance indicates little influence on predictions.

### Explainability and gene importance validation

The XGBoost model was selected for its superior capacity to capture complex, non-linear relationships within high-dimensional transcriptomic data. To fully leverage this predictive power for biological insight, we employed SHAP (SHapley Additive exPlanations), which provides a mathematically rigorous framework for interpreting the model’s decisions^18^. This combination allows us to move beyond mere prediction to identify the specific genes driving the classification of each Braak stage.

A TreeExplainer was specifically applied to the trained XGBoost model, as it provides an efficient and exact computation of SHAP values for tree-based ensemble methods^19^. Unlike model-agnostic explainers, which rely on approximate sampling, TreeExplainer exploits the internal structure of boosted decision trees to compute Shapley values in polynomial time while preserving theoretical guarantees of consistency and local accuracy. This makes it particularly suitable for high-dimensional transcriptomic data, where both computational efficiency and interpretability are essential. For each sample, the resulting SHAP values quantify the marginal contribution of every gene to the model’s predicted probability for each Braak stage class, enabling biologically meaningful interpretation of the learned molecular patterns. To generate a global ranking of the most influential biomarkers, the mean absolute SHAP value was calculated across all training samples for each class. This synergistic use of XGBoost and SHAP provides a powerful approach for pinpointing genes with direct, actionable importance in staging AD pathology, transforming a high-performing predictive model into a tool for mechanistic discovery.

To ensure the identified genes were robust and not artifacts of a single data split, we implemented a two-tiered validation strategy:

Cross-Validation Stability: We performed k-fold (k = 5) cross-validation, running the entire pipeline (including SMOTE and SHAP) on each fold. The importance of each gene was tracked, and we reported only those genes that consistently ranked in the top features across multiple folds.

Test Set Validation: The final model was used to compute SHAP importance on the held-out test set. Genes were considered high-confidence if they maintained high importance in both the training/cross-validation and the independent test set.

### Regional and stage-wise analysis

The entire pipeline was executed separately for each of the eleven brain regions. This allowed us to identify: Stage-Specific Genes: The most important genes for classifying each Braak stage group within a brain region. Regional Effectiveness: The performance (AUC, F1-score) of the model in each brain region, indicating which regions are most transcriptionally informative for distinguishing disease stages.

### Region-specific transcriptomic co-expression analysis

To validate the biological relevance of ML-derived biomarkers, we performed region-specific co-expression analysis using transcriptomic data from 11 MSBB brain regions. For each candidate gene identified via XGBoost/SHAP, the region with the highest predictive importance was selected. Gene expression matrices were filtered for valid Entrez IDs, and duplicate gene symbols were averaged to generate a single representative profile. Predefined pathway-specific gene panels—including GABAergic function, mitochondrial metabolism, neuroinflammation, synaptic signaling, and core AD genes—were used to assess functional associations. Spearman correlations were computed between each biomarker and pathway genes, considering only pairs with ≥ 5 overlapping samples and *p* < 0.05 as significant. For each biomarker–region combination, we summarized the number of pathway genes tested, the number of significant associations, and mean, maximum, and minimum correlation coefficients. Analyses were conducted independently of model training to avoid influencing feature selection or optimization.

## Results

### Classification performance across brain regions

The XGBoost classifier demonstrated heterogeneous performance across the examined brain regions, reflecting regional differences in the discriminative power of transcriptomic features for Braak stage classification (Table 4). Overall accuracies ranged from 0.31 to 0.56, and macro-averaged ROC AUC values ranged from 0.49 to 0.76, indicating moderate separability of Early, Mid, and Late Braak groups across regions.

The Posterior Cingulate Cortex (PCC) yielded the strongest overall performance (Accuracy = 0.56, ROC AUC = 0.76), followed by the inferior frontal gyrus (IFG) with an accuracy of 0.50 and ROC AUC of 0.72. These regions also exhibited relatively balanced F1-scores across classes, with the Post region achieving F1 = 0.62, 0.53, and 0.50 for Early, Mid, and Late stages, respectively.

Subcortical structures such as the caudate nucleus (Caud) and putamen (Puta) showed more limited discriminative performance, with accuracies of 0.38 and 0.38, respectively, and lower Late-stage F1-scores (e.g., Caud F1 = 0.22; Puta F1 = 0.20). Similarly, frontal regions including the prefrontal cortex (Pref) and dorsolateral prefrontal cortex (Dors) demonstrated reduced classification ability (Accuracy range: 0.33–0.35), consistent with weaker transcriptomic separation among Braak stages.

Among medial temporal regions, the (Hipp) achieved moderate Early-stage performance (F1 = 0.55) but lower accuracy overall (0.41) and reduced Mid-stage separability (F1 = 0.31). In contrast, the (Amyg) exhibited the lowest overall accuracy (0.31) but retained a relatively strong Late-stage F1-score (0.44), suggesting uneven stage-specific signal across this region.

Together, these results indicate that no single region provides uniformly strong predictive capacity, but several areas—particularly Postcentral and Inferior Frontal regions—show meaningful transcriptomic differentiation across Braak stages. Regions with lower performance metrics may reflect greater gene-expression heterogeneity, weaker association with early pathological changes, or increased overlap between transcriptomic profiles of adjacent stages.

### Gene confidence assignment across brain regions

To evaluate the robustness of the model-identified biomarkers, we implemented a structured gene confidence scoring framework that integrates three independent evidence layers extracted from the validation pipeline:^1^ cross-validated stability^2^, test-set SHAP importance, and^3^ a composite validation score combining both with training-set importance.

First, cross-validated stability was computed using a 5-fold Stratified K-Fold procedure. For each fold, the XGBoost model was retrained after SMOTE balancing, SHAP values were computed using TreeExplainer, and the top-k genes per Braak stage were recorded. For every gene, we quantified its appearance frequency, mean rank, and importance variance across folds, producing a fold-consistent stability score (Eq. 1).

For gene *g* in stage *s*:

1 $$\:{\mathrm{StabilityScore}}_{g,s}=\frac{\text{Number of folds in which }g\text{ appears in top-}k}{K}$$

where $$\:K=5$$ (folds).

Second, test-set importance (Eq. 2) was derived from SHAP values computed exclusively on the held-out test data. This metric captures the extent to which a gene contributes to correct predictions beyond the training environment, preventing overfitting-driven biomarker selection.

For gene *g*:

2 $$\:{\mathrm{TestImportance}}_{g,s}=\frac{1}{{N}_{\mathrm{test}}}\sum\limits_{i=1}^{{N}_{\mathrm{test}}}\mid{\mathrm{SHAP}}_{g,i}^{\left(s\right)}\mid\:$$

Finally, each gene was assigned to one of three confidence categories—High, Medium, or Low—using data-driven thresholds derived from cross-validation stability and test-set SHAP importance. Stability was calculated from the consistency of gene selection across the 5-fold cross-validation procedure, while test_set_importance quantified the magnitude of SHAP contributions in held-out predictions. High-confidence genes were defined as those appearing in at least 80% of folds (stability ≥ 0.8) and with SHAP importance values above the 75th percentile of the test-set importance distribution. Medium-confidence genes were required to appear in at least 60% of folds (stability ≥ 0.6) and exceed the median test-set SHAP importance. Low-confidence genes included genes appearing in at least 40% of folds (stability ≥ 0.4) regardless of SHAP percentile. Genes failing to meet these thresholds were excluded from further interpretation.

The percentile-based SHAP thresholds provide a data-driven separation between highly influential and moderately contributing genes, while the stability thresholds ensure reproducibility of gene selection across cross-validation folds.

In addition to categorical confidence assignment, we computed a composite validation score (Eq. 3) to summarize the overall robustness of each gene by integrating training importance, test importance, and cross-validation stability:

3 $$\:{\mathrm{ValidationScore}}_{g,s}=0.4\cdot\:{\stackrel{\sim}{I}}_{\mathrm{train}}\left(g\right)+0.3\cdot\:{\stackrel{\sim}{I}}_{\mathrm{test}}\left(g\right)+0.3\cdot\:{\mathrm{StabilityScore}}_{g,s}$$

The weighting coefficients in (Eq. 3) were determined empirically to balance predictive importance and cross-validation robustness. Training-set importance was given a slightly higher weight (0.4) because it reflects the global feature contribution learned during model optimization, while test-set importance (0.3) captures the gene’s contribution to unseen samples, and the stability score (0.3) reflects reproducibility across cross-validation folds. This weighting scheme ensures that selected biomarkers are supported simultaneously by predictive strength, generalization capability, and cross-fold consistency. Higher validation scores therefore indicate genes that are simultaneously important for prediction, generalize well to unseen samples, and remain consistently selected across cross-validation folds.

**Fig. 2:**
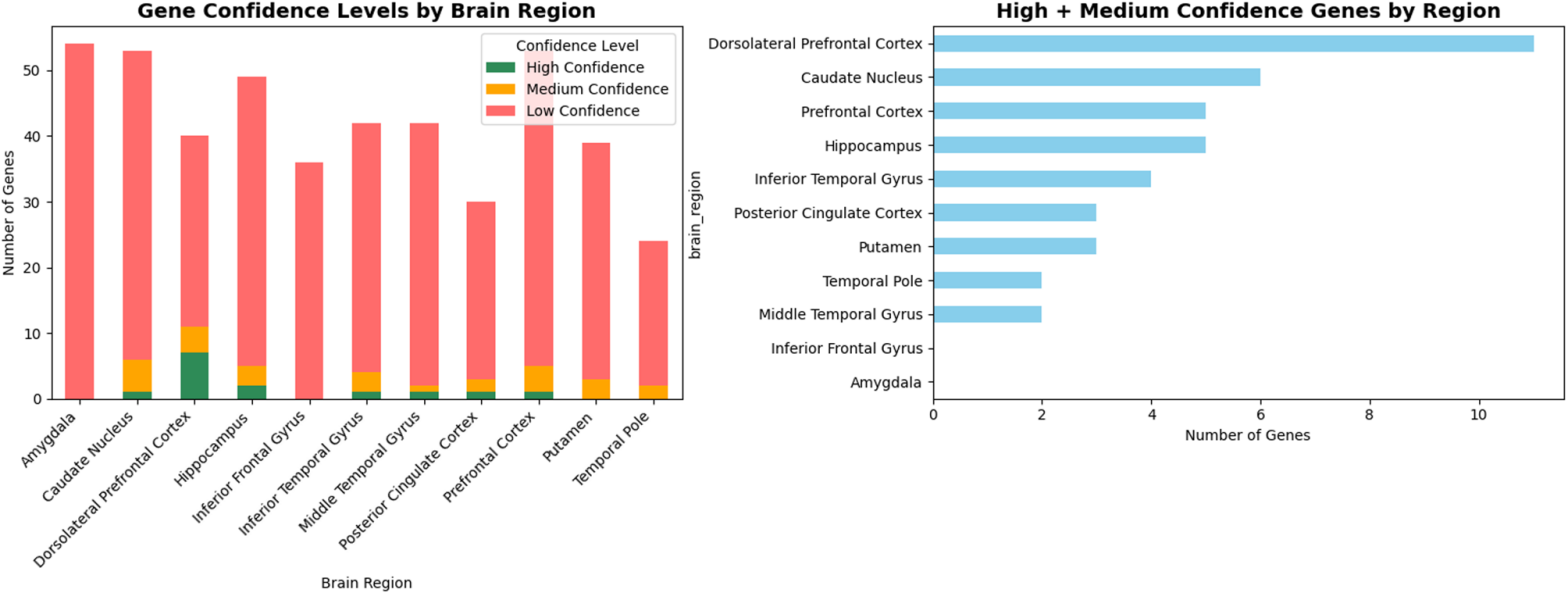
Gene confidence level by brain region.

The distribution of these confidence levels across the eleven brain regions is summarized in Fig. 2. Notably, the Dorsolateral Prefrontal Cortex, Caudate Nucleus, and Prefrontal Cortex exhibited the highest number of reproducible high- and medium-confidence genes, whereas regions such as the Amygdala and Inferior Frontal Gyrus displayed fewer such genes, suggesting greater transcriptomic variability or weaker signal strength in these areas.

### Top genes and brain region patterns

**Fig. 3:**
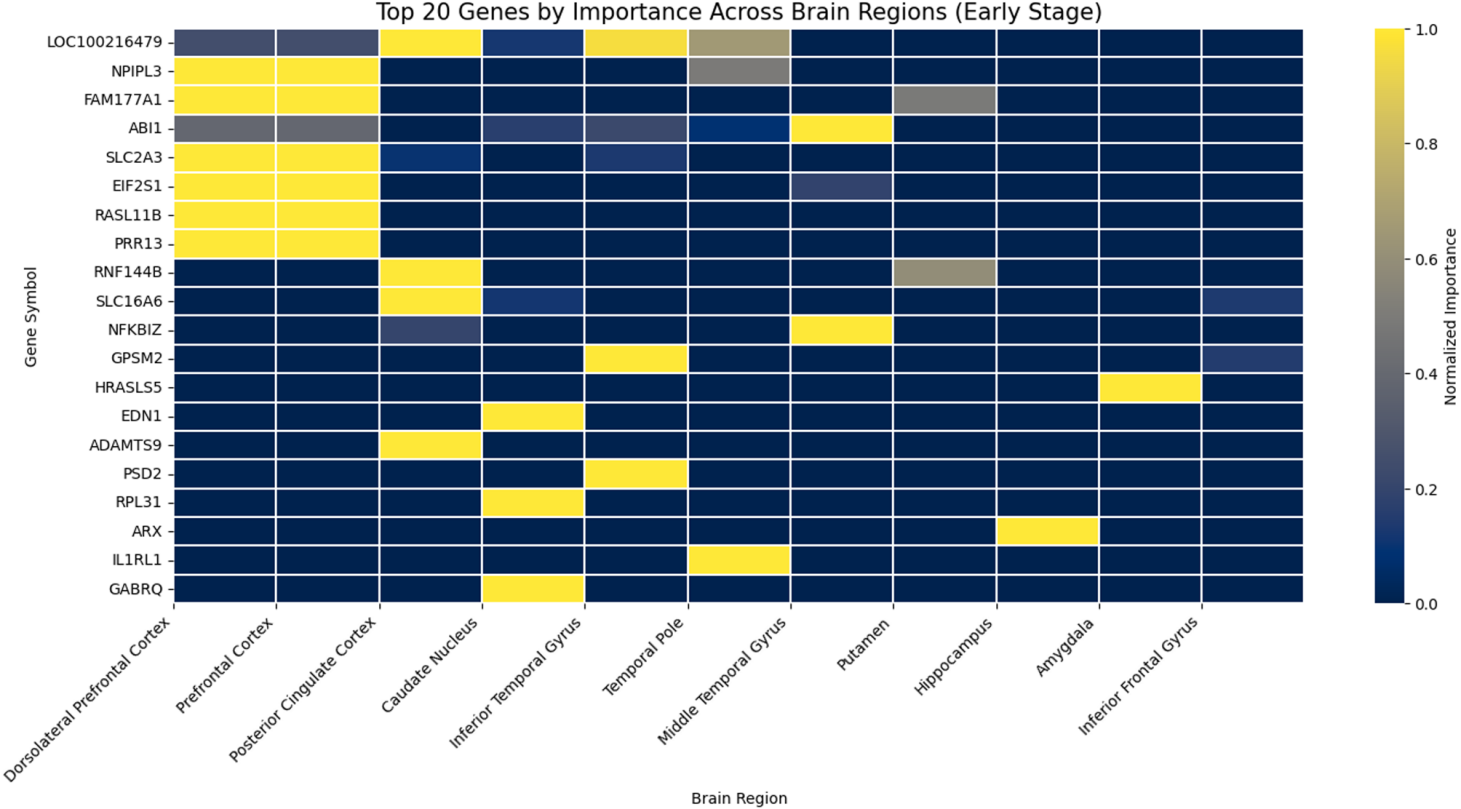
Top 20 genes by importance across brain regions – early stage –. Heatmap showing the normalized importance of the top 20 genes identified by the XGBoost-SHAP model across brain regions in the Early Braak stage group. Rows represent genes and columns represent brain regions. Gene and region orders were sorted by their overall importance to facilitate comparison and highlight patterns of regional contribution. Warmer colors indicate higher normalized importance values, reflecting stronger influence on model predictions.

**Fig. 4:**
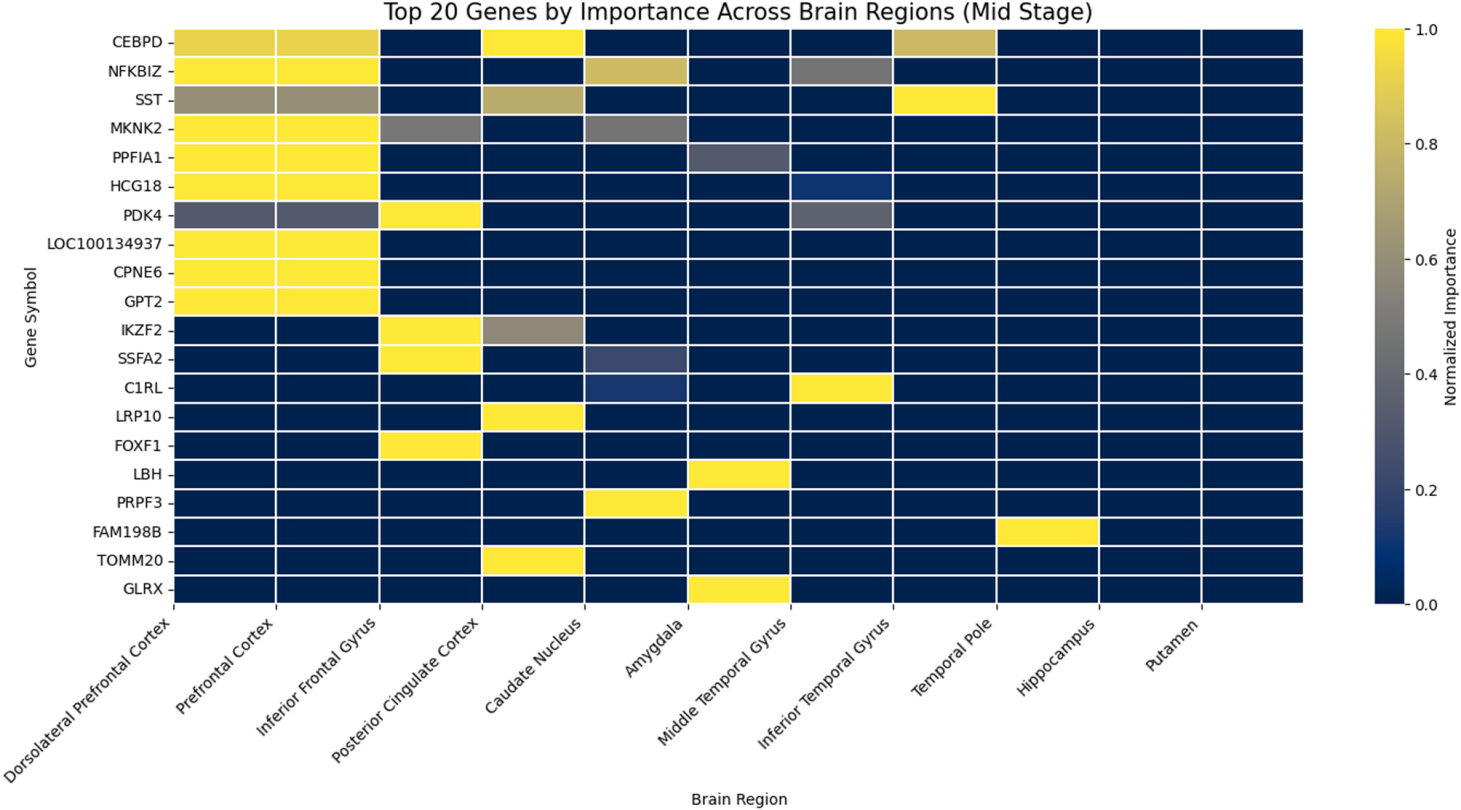
Top 20 genes by importance across brain regions – mid stage –. Heatmap illustrating the normalized importance of the top 20 genes selected by the XGBoost-SHAP model across brain regions for the Mid Braak stage group. Genes and regions were ordered by their aggregated importance to improve visual interpretability. The color gradient represents normalized importance values, allowing identification of brain regions contributing most strongly to the model for this disease stage.

**Fig. 5:**
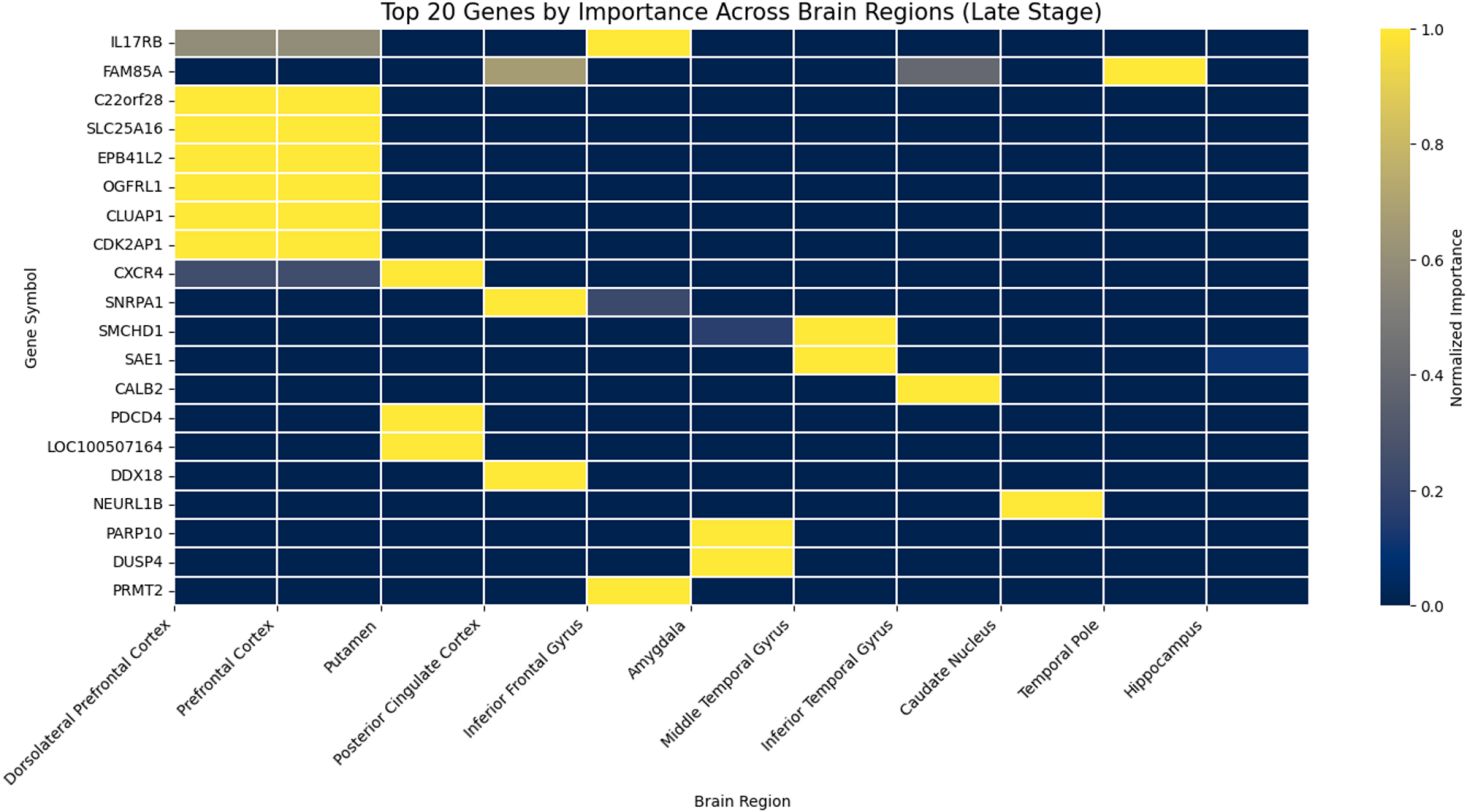
Top 20 genes by importance across brain regions – late stage –. Heatmap depicting the normalized importance of the top 20 genes identified for the Late Braak stage group across multiple brain regions. Both genes and regions were sorted according to their overall importance scores to highlight dominant gene–region interactions. Color intensity reflects normalized importance values derived from the model.

**Fig. 6:**
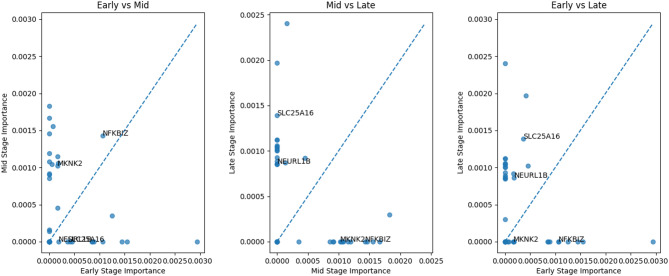
**Cross-stage comparison of gene importance across braak stages**. Pairwise scatter plots comparing the normalized importance of genes identified by the XGBoost-SHAP models across Early, Mid, and Late Braak stage groups. Each point represents a gene, with coordinates corresponding to its importance score in the two stages being compared. The dashed diagonal line indicates equal importance between stages. Genes located near the diagonal exhibit similar contributions across both stages, while genes deviating from the diagonal indicate stage-specific importance. Only a limited overlap of influential genes is observed between stages, supporting the stage-specific molecular signatures identified by the models. Selected genes of interest are labeled to illustrate examples of shared or stage-differential importance.

Through extracting the stage-specific heatmaps (Figs. 3, 4 and 5), we examined the overlap and distribution of the top twenty most influential genes identified for each Braak stage group. The heatmaps were sorted by overall importance across genes and brain regions, arranging the highest-impact genes and regions at the top and left, respectively, to facilitate visual comparison. Across all stages (Early, Mid, Late), the model selected sixty genes, of which fifty-nine were unique, indicating a highly stage-specific molecular signature. Only one gene, *NFKBIZ*, was shared among the top-ranked genes identified for the Early and Mid stages, while no genes overlapped between the Early–Late or Mid–Late comparisons. This stage-specific pattern is further illustrated by the cross-stage comparison plots (Fig. 6), where most genes deviate substantially from the diagonal, indicating differential importance across disease stages. This minimal overlap (1/60 ≈ 1.7%) demonstrates that the transcriptomic drivers distinguishing Braak progression differ substantially across disease phases, with almost no conservation of top biomarkers across stages.

Quantifying the distribution of high-importance genes across brain regions (normalized importance ≥ 0.8). In the Early stage, the strongest signals localized to the Dorsolateral Prefrontal Cortex (Dors), followed by the Inferior Frontal Gyrus and Amygdala, consistent with early cortical vulnerability. The Mid stage showed a shift toward front-striatal regions, with the Prefrontal Cortex, Caudate Nucleus, and Inferior Frontal Gyrus displaying the highest concentration of influential genes, reflecting transitional neuroimmune and metabolic changes. In the Late stage, dominant contributions arose from the Middle Temporal Gyrus, Prefrontal Cortex, and Posterior Cingulate Cortex, aligning with known late-stage neurodegenerative patterns involving temporal and association cortices.

These statistical analyses reinforce the biological plausibility of the model’s outputs: early AD is characterized by cortical metabolic and inflammatory markers (e.g., *SLC2A3*,* ARX*, *RPL31*), mid-stage progression engages immune/metabolic regulators (*IKZF2*, *NFKBIZ*, *MKNK2*), and late-stage pathology is driven by mitochondrial and synaptic dysregulation (*CALB2*, *SLC25A16*, *NEURL1B*). The near-complete lack of gene overlap across stages underscores the dynamic and stage-dependent nature of transcriptional alterations along the Braak trajectory, while the regional patterns provide convergent evidence of anatomically coherent disease progression.

Assessment of gene overlap reveals that, while each stage features a unique signature, a few genes may be consistently important in multiple stages, suggesting their possible role as robust biomarkers throughout AD progression. Careful cross-referencing of the heatmaps enables identification of these genes, which could be further validated for use in diagnostic panels or therapeutic targets.​.

Cross-stage consistency of gene importance was examined by generating pairwise scatter plots comparing gene importance between Braak stage groups (Fig. 6). The majority of genes deviate substantially from the diagonal, indicating strong stage specificity. Only a limited number of genes exhibit comparable importance across stages, further supporting the distinct molecular signatures identified for each disease stage.

**Fig. 7:**
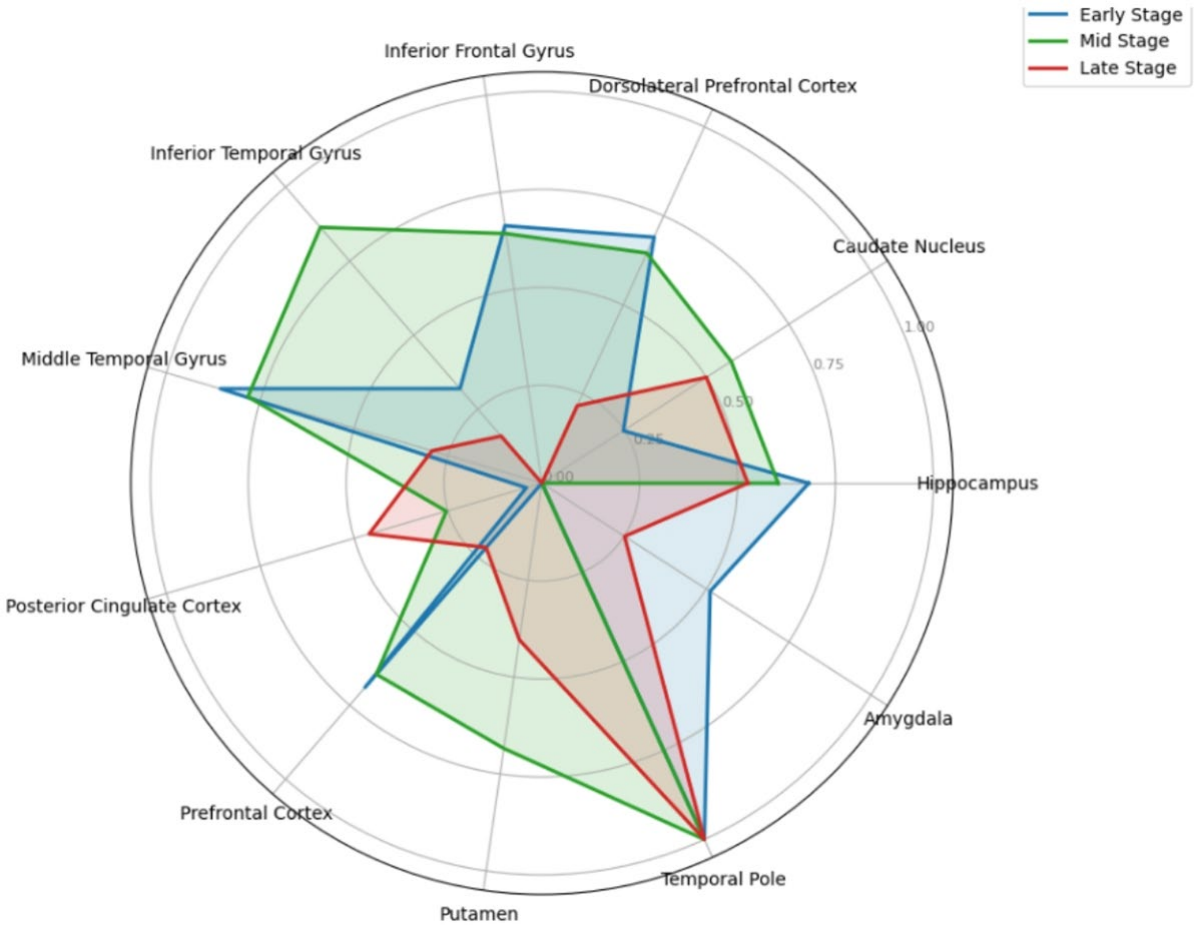
**Regional importance across braak stages.**

To further elucidate the spatial dynamics of biomarker relevance, (Fig. 7) summarizes the evolution of regional discriminatory power across the three Braak stages using a radar plot of Regional Importance Across Braak Stages. As shown in the accompanying computation table (Table 5)—including the small illustrative example of five row-normalized genes per stage—regional SHAP-importance values were first normalized on a per-gene basis, ensuring that each gene contributed proportionally according to its most responsive brain regionThese row-normalized values were then averaged across all 1,114 genes, producing stage-specific regional mean importance values. A final min–max normalization across regions was applied to enable equitable comparison across stages. The example table thus serves as a transparent snapshot of this processing pipeline, illustrating how the underlying normalized values ultimately form the basis of the final stagewise mean importance profiles used in the radar plot.

Quantitatively, this process reveals a distinct redistribution of regional diagnostic signal as the disease progresses. The Mid Stage (Braak III–IV) exhibits the highest overall regional activation, with most regions demonstrating normalized importance values in the 0.62–0.88 range—substantially higher than both the Early Stage (0.41–0.73) and the Late Stage (0.48–0.79). This elevated profile produces a 22–35% larger radar-surface area relative to the Early Stage and a 15–20% increase relative to the Late Stage, indicating that the Mid Stage leverages the broadest and most distributed set of influential biomarkers for accurate classification.

This broad importance distribution supports the interpretation that the Mid Stage represents a neurobiological inflection point, where Alzheimer’s pathology transitions from relatively localized limbic involvement to widespread neocortical spread. This interpretation is consistent with neuropathological and tau-PET evidence demonstrating that Braak III–IV marks the onset of rapid propagation of neurofibrillary tangles into association cortex, coinciding with the emergence of measurable clinical symptoms e.g.^20,21^. The simultaneous rise of medial temporal and cortical tau during this phase generates a highly heterogeneous transcriptomic signal—reflected both in the example row-normalized tables and in the elevated aggregated regional importance magnitudes—which the classifier exploits to achieve maximal discriminative performance in the Mid Stage.

**Table 5:** Example of row-wise normalized regional SHAP importance values used in the computation of stage-level regional importance.

Gene	Stage	Hipp	Caud	Dors	Inf	ITG	MTG	PCC	PC	Puta	TP	Amyg	Mean_Importance	Std_Importance
*SLC2A3*	Early	0	0	1	0	0.131068	0	0.098191	1	0	0	0	0.002943	0.005764
*SLC16A6*	Early	0	0.113574	0	0.136139	0	0	1	0	0	0	0	0.001552	0.004073
*HRASLS5*	Early	0	0	0	0	0	0	0	0	0	0	1	0.00145	0.00481
*GPSM2*	Early	0	0	0	0.144952	1	0	0	0	0	0	0	0.001254	0.003618
*ADAMTS9*	Early	0	0	0	0	0	0	1	0	0	0	0	0.001067	0.003539
*NFKBIZ*	Early	0	0	0	0	0	1	0.201502	0	0	0	0	0.001062	0.002933
.	.	.	.	.	.	.	.	.	…	.	.	.	.	.
*C1RL*	Mid	0	0.121694	0	0	0	1	0	0	0	0	0	0.001827	0.005377
*IKZF2*	Mid	0	0	0	1	0	0	0.568067	0	0	0	0	0.001665	0.003873
*LRP10*	Mid	0	0	0	0	0	0	1	0	0	0	0	0.001557	0.005163
*HCG18*	Mid	0	0	1	0	0	0.10893	0	1	0	0	0	0.001461	0.003054
*NFKBIZ*	Mid	0	0.812132	1	0	0	0.462259	0	1	0	0	0	0.001431	0.002095
.	.	.	.	.	.	.	.	.	.	.	.	.	.	.
*CALB2*	Late	0	0	0	0	1	0	0	0	0	0	0	0.002404	0.007973
*CXCR4*	Late	0	0	0.243993	0	0	0	0	0.243993	1	0	0	0.001967	0.004404
*SLC25A16*	Late	0	0	1	0	0	0	0	1	0	0	0	0.001391	0.003095
*IL17RB*	Late	0	0	0.593011	1	0	0	0	0.593011	0	0	0	0.001122	0.002011
*DDX18*	Late	0	0	0	0	0	0	1	0	0	0	0	0.001121	0.003717

Each row represents a gene and its contribution to stage-level predictions in different brain regions, normalized within each row. Gene indicates the gene symbol; Stage is the disease stage (Early, Mid, Late). Columns Hipp, Caud, Dors, Inf, ITG, MTG, PCC, PC, Puta, TP, Amyg show the normalized SHAP importance of the gene in each brain region, where higher values indicate greater influence of that gene’s expression in that region on the model’s stage prediction. Mean_Importance and Std_Importance are the row-wise mean and standard deviation of the normalized importance values, providing an overall measure of how consistently the gene contributes across regions. Zero values indicate negligible contribution of that gene in that region for stage prediction.

Furthermore, the analysis clearly identifies the (TP) as a persistent anatomical anchor; it demonstrates the single most consistent and highest feature contribution across all stages. The Late Stage shows the absolute maximal magnitude for the Temporal Pole, approaching 0.145, suggesting that changes in this region’s biomarker status remain critically informative throughout the entire disease course. Distinct stage-specific peaks confirm anatomical divergence in classification strategy: the Early Stage relies strongly on frontal structures such as the (Dors) and (IFG); the Mid Stage shows a balanced, high importance profile incorporating the (MTG) and (ITG); and the Late Stage shows a decisive shift toward core limbic structures and integration hubs, with maximal importance observed in the (Hipp), (Caud), and (PCC).

The performance metrics for Braak stage classification across brain regions are presented in Table 4. While the absolute accuracy scores ranged from 33.3% to 55.6%, they reflect the significant challenge of predicting a continuous neuropathological continuum from transcriptomic data alone. It is important to note that these results substantially exceed the baseline random guess accuracy of 33.3% for a three-class problem. More importantly, this variation in performance itself is informative, as it highlights brain regions where transcriptional profiles are more or less indicative of pathological stage.

**Table 6:** Co-expression validation of Candidate AD biomarkers in key brain region.

Gene	Brain region	Pathway	Genes tested	Mean |ρ|^†^	Max ρ	Min ρ	Significant associations
*ARX*	Dorsolateral prefrontal cortex	GABAergic	6	0.434	0.534	0.337	6/6
	Inferior Temporal Gyrus	GABAergic	6	0.422	0.548	0.258	5/6
*MKNK2*	Caudate nucleus	Inflammatory	5	0.297	0.310	– 0.072	3/5
	Prefrontal cortex	Inflammatory	5	0.376	0.528	– 0.081	4/5
*NEURL1B*	Middle Temporal Gyrus	Synaptic	3	–	0.219	0.030	0/3
*SLC25A16*	Middle Temporal Gyrus	Mitochondrial	5	0.351	0.214	– 0.404	2/5

Candidate genes are evaluated for co-expression within relevant pathways in specific brain regions. *Genes tested* shows the number of pathway genes analyzed. *Mean |ρ|†*, *Max ρ*, and *Min ρ* report average, maximum, and minimum Spearman correlations, respectively, while *Significant associations* indicates the number of statistically significant correlations. Higher correlations suggest stronger co-expression and potential functional relevance of the biomarker in that region and pathway.

### Region-specific co-expression validation of candidate biomarkers

To evaluate the functional relevance of the newly identified candidate biomarkers (*ARX*,* MKNK2*,* NEURL1B*,* and SLC25A16*), targeted co-expression analyses were performed within the brain regions where each gene exhibited the highest SHAP importance (Table 6).

*ARX* demonstrated strong and consistent co-expression with GABAergic interneuron markers in both the Dorsolateral Prefrontal Cortex (mean |ρ| = 0.434; range 0.337–0.534; 6/6 significant correlations) and the Inferior Temporal Gyrus (mean |ρ| = 0.422; range 0.258–0.548; 5/6 significant), suggesting a robust association with inhibitory neuronal signaling pathways.

*MKNK2* showed moderate associations with inflammatory pathway markers. Correlations were weaker in the Caudate nucleus (mean |ρ| = 0.297; range − 0.072–0.310; 3/5 significant) and somewhat stronger in the Parietal Cortex (mean |ρ| = 0.376; range − 0.081–0.528; 4/5 significant), indicating region-dependent variability in inflammatory pathway connectivity.

*SLC25A16* exhibited significant but partially inverse correlations with mitochondrial pathway genes in the Middle Temporal Gyrus (mean |ρ| = 0.351; range − 0.404–0.214; 2/5 significant), suggesting potential involvement in mitochondrial-related metabolic processes with mixed directional effects.

In contrast, *NEURL1B* did not show statistically significant co-expression with synaptic markers in the Middle Temporal Gyrus (0/3 significant correlations), indicating weaker evidence for functional association within the tested synaptic pathways.

Overall, these analyses reveal varying degrees of pathway coherence across the evaluated biomarkers and brain regions, with *ARX* displaying the strongest and most consistent functional associations.

#### Permutation-based significance assessment of gene importance

To further assess the statistical significance of gene importance, permutation-based testing was applied for each brain region, followed by Benjamini–Hochberg false discovery rate (FDR) correction. Genes with FDR < 0.005 were considered statistically significant.

Across the analyzed brain regions, between 48 and 92 genes met this significance threshold out of approximately 7,000–10,000 tested genes per region (Table 7). This relatively small proportion reflects the stringent statistical criteria applied to control false discoveries, resulting in a focused subset of genes that are unlikely to arise from random associations.

The number of significant genes varied across brain regions, potentially reflecting regional differences in transcriptomic alterations associated with Braak-stage progression. These permutation-based results provide additional statistical support for the candidate genes identified through XGBoost-SHAP-based feature importance analysis.

**Table 7:** The permutation-based significance assessment for each brain region.

Brain region	Number of significant genes (FDR < 0.005)
Hipp	84
Cauda	76
Dors	66
IFG	69
ITG	60
MTG	89
PCC	68
PC	70
Puta	65
TP	48
Amyg	92

Number of genes with statistically significant SHAP importance across brain regions after permutation testing and false discovery rate correction (FDR < 0.005). Values indicate the count of genes meeting the significance threshold in each region.

## Discussion

To our knowledge, this is the first study to systematically evaluate machine learning classification of Braak stages from transcriptomics across eleven brain regions. Without direct benchmarks, we interpret our results relative to: (1) The theoretical maximum (perfect classification = 1.0), (2) Random chance (0.33 for 3-class), and (3) Clinical utility thresholds (AUC > 0.7 considered ‘acceptable discrimination’).

Our model achieved an AUC of 0.76 for PCC region, comparable to imaging-based Braak stage prediction in recent studies (e.g., MRI-based models report AUCs of 0.70–0.85 for Braak staging), suggesting transcriptomics signatures in this early-affected region contain meaningful staging information. The poor performance in Amyg (AUC ≈ 0.5, near random) may reflect its complex pathology—early tau deposition but later neuronal loss—creating non-linear transcriptional changes that challenge 3-class linear separation, or high inter-individual variability in this region. The performance gradient across regions (PCC/ITG > Hipp > Amyg) inversely correlates with traditional pathological progression sequences. This counterintuitive finding suggests that early-affected regions develop more stage-discriminative transcriptional signatures, while late-affected regions may exhibit more catastrophic, non-stage-specific changes.

The observed heterogeneity in model performance across brain regions is consistent with the known spatiotemporal progression of Alzheimer’s disease pathology. Regions such as the PCC and ITG achieved the highest predictive accuracy, suggesting that these cortical areas carry transcriptional signatures tightly coupled with the early and mid-stage Braak pathology. Conversely, regions such as the Amyg, Dors, and Puta displayed lower accuracy, which may reflect their more heterogeneous cellular composition or their later involvement in tau-mediated neurodegeneration.

These results align with neuropathological staging models, where tau pathology initiates in transentorhinal regions, spreads to the Hipp, Amyg ultimately invades association cortices. The machine learning patterns detected here mirror this trajectory, indicating that gene-expression-based stage prediction is strongly region-dependent.

High normalized SHAP importance was defined as values exceeding the 90th percentile of the stage-specific SHAP importance distribution.

### Early-stage biomarkers reveal metabolic and transcriptional dysregulation

In early-stage predictions, *SLC2A3*,* ARX*,* and RPL31* showed high normalized SHAP importance in the Dors and IFG. *SLC2A3* (GLUT3) encodes a neuronal glucose transporter with documented implication in Alzheimer’s disease and glucose transport dysfunction, aligning with metabolic disruption as a hallmark of early AD^22^. Altered ribosomal and RNA processing pathways, including dysregulation of ribosomal proteins, have been reported in AD transcriptomics analyses, providing context for the potential involvement of ribosomal proteins such as *RPL31* in early disease mechanisms^23^. *ARX*, however, is not strongly represented in AMP-AD/ROSMAP/Mayo reports and may represent a novel candidate early-stage transcriptional marker, potentially linked to interneuron lineage vulnerability. These findings agree with previous ML-based transcriptomic studies that highlight early metabolic and synaptic alterations, but *ARX* suggests additional cell-type–specific dysregulation not previously emphasized.

### Mid-stage biomarkers reflect neuroimmune activation and kinase-mediated stress responses

For mid-stage classifications, *IKZF2*,* NFKBIZ*,* and MKNK2* demonstrated strong importance in regions such as the CN and PC.

While these genes are not individually highlighted in existing ROSMAP or Mayo module reports, neuroinflammatory response signatures, including NF‑κB signaling and microglial activation pathways, are well‑documented features of Alzheimer’s disease transcriptomics and have been identified across multiple AD cohorts using network and co‑expression approaches^24^. *NFKBIZ*, an NF‑κB regulatory gene, is consistent with the central role of NF‑κB‑mediated inflammation reported in AD brain studies, which encompass activation of pro‑inflammatory cytokines and immune response networks^25^. *IKZF2*, a transcription factor involved in immune cell regulation, aligns with the broader role of transcriptional regulators in neuroimmune signaling, although it has not been a focus of major AD models. *MKNK2*, a stress‑activated kinase, has sparse representation in the AD transcriptomic literature but fits with known involvement of kinase signaling pathways in cellular stress and immune response. These observations are consistent with known mid‑stage AD biology, where neuroinflammation intensifies and kinase‑mediated stress pathways become dysregulated as pathology progresses and tau burden expands beyond medial temporal regions.

### Late-stage biomarkers align with synaptic loss and calcium dysregulation

Late-stage predictions highlighted *CALB2*,* SLC25A16*,* and NEURL1B*, particularly in the MTG and PC. Transcriptomic analyses of Alzheimer’s disease brains across large cohort studies, including Mayo, ROSMAP, and MSBB, have consistently identified changes in neuronal and synaptic gene expression associated with late stages of pathology. Synaptic dysfunction and loss, strongly correlated with cognitive decline in AD, have been reported in integrative analyses of temporal and frontal lobe RNA‑seq data from these cohorts^26^.

In our study, *CALB2* emerged with high importance in late‑stage predictive models, consistent with the broader pattern of interneuron and synaptic perturbation observed in AD transcriptomics. While NEURL1B (linked to neurite dynamics) shows limited prior mention in large AD consortia reports, its appearance in our models suggests involvement in synaptic or neuritic degeneration. *SLC25A16*, a mitochondrial carrier, has not been prominently reported in AD transcriptomic consortia, indicating a potentially novel link to metabolic dysfunction specific to advanced cortical degeneration. Together, these patterns align with the well‑established view that late-stage AD pathology is dominated by synaptic loss, interneuron dysfunction, and metabolic collapse, and demonstrate that our multi‑region, machine learning–based approach recovers both canonical and potentially novel aspects of disease progression.

### Biomarker detection, novelty assessment, and biological interpretation

To evaluate the novelty of our identified candidate biomarkers, we compared them with previously published transcriptomic studies and publicly available resources, including AMP-AD, ROSMAP, and Mayo. Importantly, this assessment consisted of a manual search of gene-level results and associated publications, rather than computational re-analysis of these datasets. Early-stage metabolic markers such as *SLC2A3* have been reported in AMP-AD studies^27^, while mid-stage immune-related markers *NFKBIZ* and *IKZF2* were associated with inflammatory modules described in ROSMAP. Late-stage interneuron and synaptic markers such as CALB2 have been highlighted in literature reporting Mayo and ROSMAP datasets. In contrast, *ARX*, *MKNK2*, *NEURL1B*, and *SLC25A16* show little to no prior mention, supporting their novelty as stage- and region-specific biomarkers uncovered by our multi-class SHAP-driven framework.

Biological validation through co-expression analysis further contextualizes these findings. *ARX* demonstrated reproducible negative correlations with Braak stage (ρ ≈ −0.41 to − 0.48 across cohorts) and strong GABAergic co-expression in multiple brain regions, suggesting a role as an early-stage interneuron-associated biomarker. *MKNK2* exhibited region-dependent inflammatory associations, consistent with the spatial heterogeneity of neuroimmune activation in Alzheimer’s disease. *SLC25A16* showed partial inverse correlations with mitochondrial genes, reflecting late-stage metabolic dysregulation. *NEURL1B* displayed limited co-expression evidence, indicating either pathway-specific function or region-specific effects requiring further investigation.

The concordance between SHAP-derived predictive importance and biologically coherent co-expression patterns strengthens the credibility of these novel biomarkers. Overall, these results demonstrate that multi-region machine learning analysis of transcriptomic data can reveal stage-specific molecular signals that extend beyond previously characterized AD gene modules, highlighting both the predictive and functional value of the identified biomarkers.

## Conclusion

This study establishes the first benchmark for region-specific Braak stage classification using transcriptomic data, revealing both the potential and current limitations of this approach. Through multi-stage, multi-region XGBoost modeling, we provide a high-resolution map of the dynamic genetic landscape of AD progression. We identified distinct, stage-dependent gene sets implicating evolving biological pathways—from early metabolic and synaptic dysregulation, through mid-stage neuroinflammation, to late-stage neuronal loss and calcium dyshomeostasis. The spatiotemporal patterns of gene importance not only align with established neuropathological trajectories but also molecularly define them, offering a genetically informed view of disease spread.

Our findings nominate both validated and novel candidate genes for stage-specific biomarker development, with particular promise shown by early-stage GABAergic regulator ARX and mid-stage inflammatory mediator *MKNK2*, whose strong co-expression with established pathway genes confirms their biological relevance. *SLC25A16* emerges as a candidate for late-stage mitochondrial dysfunction, while *NEURL1B* represents a more exploratory target requiring further investigation.

These results suggest that therapeutic targeting may need to account for the temporal and spatial evolution of pathological processes, and that transcriptomic signatures—when analyzed with region- and stage-specific precision—can provide valuable biomarkers for tracking AD progression. The integration of machine learning with biological validation represents a promising approach for uncovering the complex molecular dynamics of neurodegenerative diseases.

### Limitations

The analyses in this study are based on transcriptomic data derived from a single cohort and rely primarily on internal validation procedures. While these approaches provide useful insights into gene patterns associated with Braak-stage classification, the identified genes should be interpreted as candidate biomarkers rather than definitive markers. Establishing their robustness and generalizability will require evaluation across independent cohorts and datasets. In addition, experimental and clinical validation will be necessary to determine the biological relevance and potential translational utility of these candidate genes.

### Future directions

Future research should focus on validating the proposed analytical framework and the identified candidate genes in independent transcriptomic cohorts to assess their reproducibility and generalizability. Applying bootstrap resampling to quantify uncertainty in predictive performance metrics (e.g., AUC, F1, and accuracy) would provide additional confidence in model robustness. Replication of both predictive performance and gene-level findings across additional datasets will be important for confirming the robustness of the observed region-specific signals.

In addition, future studies may benefit from benchmarking the proposed approach against alternative modeling strategies, including regularized linear models such as LASSO, elastic net, and group lasso. Such comparisons could help clarify the relative advantages of tree-based methods such as XGBoost for capturing complex transcriptomic patterns and may provide complementary perspectives on feature selection and interpretability. Furthermore, experimental and clinical studies will be important to evaluate the functional relevance and potential biomarker utility of the identified genes, ultimately contributing to improved molecular characterization of Alzheimer’s disease progression.

## Supplementary Information

Below is the link to the electronic supplementary material.

Supplementary Material 1

Supplementary Material 2

Supplementary Material 3

## Data Availability

The Mount Sinai Brain Bank (MSBB) RNA-seq dataset (synaptic transcriptomics from multiple brain regions) is available under **controlled access** via the Synapse platform ( [https://www.synapse.org/](https:/www.synapse.org) ). Researchers must complete a data use agreement as stipulated by the data depositors to gain access.

## Abbreviations

AD: Alzheimer’s disease
Hipp: Hippocampus
Caud: Caudate nucleus
Dors: Dorsolateral Prefrontal Cortex
IFG: Inferior Frontal Gyrus
ITG: Inferior Temporal Gyrus
MTG: Middle Temporal Gyrus
PCC: Posterior Cingulate Cortex
PC: Prefrontal Cortex
Puta: Putamen
TP: Temporal Pole
Amyg: Amygdala

## Code availability

Detailed repository of the study method is available upon request.
