# Supplementary material for: Robust transcriptomic signatures of Alzheimer’s disease progression: validated explainable AI approach
Source: Sci Rep. 2026 May 19;16:15478. doi: 10.1038/s41598-026-47879-8 (PMC13187053; doi:10.1038/s41598-026-47879-8)
Supplement: Supplementary file 1 — Supplementary Material 1 [file 41598_2026_47879_MOESM1_ESM.docx]

**2.3.2. Model Validation and Performance Metrics**

**Accuracy**

Accuracy measures the proportion of correctly classified samples relative to the total number of samples:

$\text{Accuracy}=\frac{\text{Number of Correct Predictions}}{\text{Total Number of Samples}}$ (Eq. 1)

**F1-Score**

The F1-score is the harmonic meaning of precision and recall for each class $c$:

$\text{Precision}_{c}=\frac{TP_{c}}{TP_{c}+FP_{c}}$ (Eq. 2)$\text{Recall}_{c}=\frac{TP_{c}}{TP_{c}+FN_{c}}$ (Eq. 3)$F1_{c}=2\cdot\frac{\text{Precision}_{c}\cdot\text{Recall}_{c}}{\text{Precision}_{c}+\text{Recall}_{c}}$ (Eq. 4)

The macro-averaged F1 is then:

$F1_{\text{macro}}=\frac{1}{C}\sum_{c=1}^{C} F1_{c}$ (Eq. 5)

where $C$ is the number of classes (3 Braak stage groups).

**AUC-ROC**

The ROC curve plots the True Positive Rate (TPR) versus the False Positive Rate (FPR). For each class $c$:

$TPR_{c}=\frac{TP_{c}}{TP_{c}+FN_{c}}$ (Eq. 6)$FPR_{c}=\frac{FP_{c}}{FP_{c}+TN_{c}}$ (Eq. 7)

The AUC-ROC score is defined as:

$AUC_{c}=\int_{0}^{1} TPR_{c}\left( FPR_{c} \right)\text{ }d\left( FPR_{c} \right)$ (Eq. 8)

For multi-class classification, we report the **macro-averaged AUC**:

$AUC_{\text{macro}}=\frac{1}{C}\sum_{c=1}^{C} AUC_{c}$ (Eq. 9)
