# Supplementary material for: Robust transcriptomic signatures of Alzheimer’s disease progression: validated explainable AI approach
Source: Sci Rep. 2026 May 19;16:15478. doi: 10.1038/s41598-026-47879-8 (PMC13187053; doi:10.1038/s41598-026-47879-8)
Supplement: Supplementary file 2 — Supplementary Material 2 [file 41598_2026_47879_MOESM2_ESM.docx]

**Table 1. Summary of Related Work on Machine Learning and Transcriptomics in Alzheimer's Disease**

| Study (Reference) | Primary Focus | Data Type | ML Model | Key Limitation Addressed in Our Work |
| --- | --- | --- | --- | --- |
| (13) | Correlation of gene expression with Braak stages | Bulk RNA-seq / proteomics (Mount Sinai cohort) | Linear correlation analysis | Linear methods may miss non-linear relationships; limited predictive modeling |
| (11) | Molecular subtyping of AD; identification of subtype-specific driver genes and pathways | Bulk RNA-seq (MSBB, multiple brain regions) | Network-based ML / classifier for subtypes | Focused on molecular heterogeneity; did not predict specific neuropathological stages (Braak staging); limited stability analysis across data splits |
| (14) | Transcriptomic-based AD severity index | Bulk RNA-seq | Deep learning regression | Relies on bulk tissue; does not directly predict Braak stages; may obscure cell-type-specific signals |
| (16) | Comparative analysis of blood vs brain transcriptomic biomarkers | Blood transcriptomics | Correlation-based | Peripheral biomarkers not highly specific; no predictive modeling |
| (15) | Cognitive dysfunction prediction from regional Braak-staged amyloid-β PET | Regional PET imaging | SVR, ANN | Small sample size; lack of external validation; limited class imbalance handling; limited interpretability validation |
| (12) | AD prediction using a minimal gene signature | Bulk RNA-seq (public datasets) | Ensemble ML (Logistic Regression + SVM + Naive Bayes) | Binary classification; no multi-stage prediction; limited validation for reproducibility and stability |
| (17) | Explainable CNN for regional brain features | MRI imaging | CNN with Integrated Gradients, Grad-CAM, SmoothGrad | Limited application to transcriptomics; focuses on imaging; interpretability validation limited |
| (18) | Blood-based AD stage classification | Blood RNA-seq + clinical data | Deep learning with SMOTE | Limited to blood data; may miss full spectrum of brain pathology |
