# Supplementary material for: Robust transcriptomic signatures of Alzheimer’s disease progression: validated explainable AI approach
Source: Sci Rep. 2026 May 19;16:15478. doi: 10.1038/s41598-026-47879-8 (PMC13187053; doi:10.1038/s41598-026-47879-8)
Supplement: Supplementary file 3 — Supplementary Material 3 [file 41598_2026_47879_MOESM3_ESM.docx]

| Hipp | | Caud | | Dors | | IFG | | MTG | | PCC | | PC | | Puta | | TP | | ITG | | Amyg | | |
| --- | --- | --- | --- | --- | --- | --- | --- | --- | --- | --- | --- | --- | --- | --- | --- | --- | --- | --- | --- | --- | --- | --- |
| Accuracy | ROC AUC (Macro) | Accuracy | ROC AUC (Macro) | Accuracy | ROC AUC (Macro) | Accuracy | ROC AUC (Macro) | Accuracy | ROC AUC (Macro) | Accuracy | ROC AUC (Macro) | Accuracy | ROC AUC (Macro) | Accuracy | ROC AUC (Macro) | Accuracy | ROC AUC (Macro) | Accuracy | ROC AUC (Macro) | | Accuracy | ROC AUC (Macro) |
| 0.4118 | 0.6730 | 0.3750 | 0.7014 | 0.3333 | 0.5295 | 0.3125 | 0.6528 | 0.3889 | 0.6601 | 0.5556 | 0.7596 | 0.3529 | 0.6151 | 0.3750 | 0.5338 | 0.5000 | 0.6898 | 0.5000 | 0.7170 | | 0.3125 | 0.4939 |
| 0.3529 | 0.6785 | 0.3750 | 0.6847 | 0.33333 | 0.5253 | 0.3125 | 0.6528 | 0.4444 | 0.6546 | 0.5555 | 0.7596 | 0.3529 | 0.6151 | 0.3750 | 0.5338 | 0.5000 | 0.6852 | 0.500 | 0.7169 | | 0.3125 | 0.4939 |
| 0.3529 | 0.6785 | 0.3750 | 0.6847 | 0.3333 | 0.5253 | 0.3125 | 0.52532 | 0.4444 | 0.65465 | 0.5556 | 0.7596 | 0.3529 | 0.6150 | 0.3750 | 0.5337 | 0.500 | 0.6851 | 0.500 | 0.7170 | | 0.3125 | 0.4939 |

**Table : Model Performance Metrics Across Brain Regions (3 repeated runs)**
